# Book Reviews

**Published:** 1827-10

**Authors:** 


### CRITICAL ANALYSES.

Quæ laudanda forent, et quæ culpanda, vicissim

Illa, prius, cretâ; mox hæc, carbone, *notamus*.—Persius.

*A Treatise on the Diseases of Females*. By Wm. P. Dewees, M.D. Adjunct Professor of Midwifery in the University of Pennsylvania, &c. &c.—8vo. pp. 557. Philadelphia, 1826.

Although the present work is not presented to us as a compilation, it is evident, from the general nature of its contents, that the author must have derived considerable assistance from the labours of previous writers upon the same subjects. His talents and long experience, however, peculiarly fit him for the skilful separation of the corn from the chaff in the productions of other authors, and also for the addition of much valuable information from his own resources. We could not but smile at the transatlantic bluntness of the advertisement to the volume before us. "The necessity of the work is acknowledged, but the difficulty of executing it can only be known to him who undertakes it. This will plead with the liberal for moderation in criticism, though it may be no exteuation to those of a contrary feeling. From the observations of the former we hope to profit, should any such honour the work with their notice; and from the latter we will not flinch, however severe may be the castigation, as we know it is much easier to find fault than to excel." We like the independent spirit which animates our veteran brother, and infinitely prefer it to the servile and supplicating tone which pervades the introductory pages of so many works. It is neither necessary nor judicious for an author to throw out a sop to every critical Cerberus.

The great variety of subjects discussed by the author will prevent us from following him step by step. We must assume the privilege of dwelling only upon those parts which appear to us the most interesting, and the notice of which will be amply sufficient to enable our readers to form an estimate of the value of the whole.

The first chapter takes into consideration *the Peculiarities of the Female System*. The anatomical and physiological distinctions between the sexes are briefly described. Respecting the influence of the uterus over the female constitution, Dr. Dewees entertains opinions which are worthy of notice. By some this organ was formerly supposed to possess a separate and peculiar life, having its own mode of existence, and being totally independent of the laws which govern the other portions of the system. Sydenham, Cullen, Good, and many others, have also given extensive powers to the uterus. As Dr. Dewees, however, has not witnessed any decided instances of this great influence of the uterus in the production of disease, he feels justified in entering his protest against it. He thus states his opinions :

"1st. We have ever found the unoccupied uterus to be one of great passiveness when in a state of perfect health; and that, so long as it preserved this condition, it manifested no agency in the production of disease, or in modifying it, if present. Thus fever, inflammation either local or general, or spasm, has never appeared to us to derive advantage, or suffer inconvenience, from the influence of this organ.

"2d. That, when in a state of disease, we have found several parts of the body sympathise with the uterus,—as the stomach, the head, the breasts, &c.; but precisely the same thing can be said of other parts of the body, for neither of which is such influence admitted as is claimed for the uterus. Thus the brain, the stomach, the kidneys, the liver, &c. when in a diseased state, will have particular parts deranged, by a sympathetic influence; yet it has never been asserted that either of these parts had at any other time, or under any other circumstance but disease, any agency in producing or modifying disease in any other portion of the body.

"3d. While the uterus is performing one of its functional duties,—namely, forming the menstrual blood; when it is known to be in a state of excitement, and decidedly engorged with blood, a time when it would most likely exert an influence, if it really possessed any, we never find this organ betraying a power over other parts, so long as the functional process is carried on healthfully; and that during this period we have hitherto not been able to detect the slightest influence over any disease that may have been present in the system; nor has it ever made us vary a prescription or modify a treatment.

"4th. That when the menstruous function is performed with pain and difficulty, other portions of the system are found to suffer from sympathy; but in no greater degree than these very parts have been known to suffer when some other organ was the source of irritation. In dysmenorrhœa, we have known the back and stomach suffer severely,—the first by pretty intense pain, and the second by severe vomiting; but we have seen the same consequences attend an irritated kidney, or an inflamed neck of the bladder.

"5th. That when this organ is labouring under severe disease, as inflammation, schirrus, or cancer; where all its ordinary functions are either deranged, perverted, or suspended, and this for a long period together, we do not find that it involves the system in any severer penalties than any other equally important viscus would do under similar circumstances.

"6th. That when its functional powers are irregularly and imperfectly performed, or altogether suspended, by some derangement or power, if the general health suffers from this cause, it is not because the uterus has any superior power to effect this, but because one of the links is broken, (and we are willing to admit it to be an important link,) whereby the chain of healthy functions is maintained. A similar condition of any of the other viscera would be followed by the same consequences.

"7th. That when the uterus is impregnated, various other portions of the system are deranged, in consequence of their strong sympathy with this organ; but even here the complaints are not *sui generis*, for every one of them can be, and have been very often, similated from other causes. The whole phenomena of impregnation are so well understood as not to require reciting; but has not almost every body witnessed the whole train of these morbid sympathies to arise from very different causes?" (P 23.)

Without reference to opinions which have long been abandoned, we believe that no opposition will be found to the first of the above propositions. In order that the uterus should exert a prejudicial influence upon the health of the female, the uterus must itself be in an unhealthy condition. It is true also that the derangement of any other organ may produce a variety of general symptoms, arising from its sympathetic influence upon the constitution, as well as derangement of the uterus. But, when the uterus is deranged, either in function or structure, the constitutional effects are usually very different from those which arise from the disturbance of any other viscus. The third axiom appears to us to be perfectly correct, and it deserves attention, inasmuch as former doctrines still maintain a partial influence, as it is yet thought by some practitioners that the menstrual period is one which frequently demands either a complete alteration, or at least a decided modification in the treatment required for any existing disease. In brief, the author is of opinion that the uterus ranks in the first order of viscera; that its health is every way important to the general health of the system; but that it does not exert any particular power over other portions of the body more than any other important viscus would, under the same circumstances,—namely, of disease. That its influence is greatest while performing or giving evidence of its best state of health,—namely, during gestation.

We might perhaps have expected, in a systematic work upon the Diseases of Women, that, in this introductory chapter, the author would have descanted upon the moral peculiarities of the female sex, which undoubtedly have much influence in modifying, and perhaps even in producing some diseases which are peculiar to them. This part of the subject, however, is not touched upon.

The chapter on *Menstruation* will be consulted with advantage by the student. It contains many highly judicious remarks, and very satisfactory refutations of different ridiculous doctrines upon this supposed mysterious subject, that have at various periods "lived their little day." We are told by very respectable authority, that impregnation has taken place before the commencement, and after the final cessation, of the menses. The explanation of this apparent exception to the general law, is not difficult. It has been taken for granted, that because a coloured fluid was not observed, that the uterus had not assumed the menstrual action, or did not continue it. "Now it is admitted by all practical men who have paid attention to this subject, to be a fact of no very rare occurrence for the menstrual evacuation to be serous for several periods before the menstrual blood, properly so called, shows itself, more decidedly to mark the establishment of this process. This is especially the case with those who have this discharge to commence early. The error is not less liable to be made in those rare instances of impregnation after the final cessation is supposed to have taken place; for it has been found in several well-attested instances of pregnancy at advanced periods of life, that an effort had been made by the system to restore the catamenial flux by a periodical serous discharge.

The seventh chapter is occupied by the consideration of *Deranged Menstruation.* We find the author thinks very highly of the Tr. Cantharidis and Decoction of Madder, as emmenagogues. Of the latter medicine we know nothing from personal experience: we believe, however, that in this country the power assigned to it by Dr. Dewees is generally doubted. We may remark also that the most contradictory opinions have been given respecting the effects of this medicine: Dr. Home* thought highly of it in cases of *obstructed* menstruation; while Dr. Parr† declares that it produced a cure in *excessive* menstruation, but that in the former disease it effected no change whatever. Dr. Cullen‡ tells us that, when given freely to brutes, the madder always deranges them very considerably, and appears hurtful to the system. —The former (viz. cantharides) we have frequently given in various doses, but never with any decided effect, either in

* Clinical Experiments, Histories, &c.—8vo. 1780.

† Med. Dict. p. 524.

‡ Mat. Med.

promoting the menstrual discharge or in correcting that of leucorrhœa, for which also our author considers it highly serviceable.

In our notice of Dr. HALL's recent work upon Female Diseases, we deprecated the popular plan of giving "forcing medicines," without reference to the general health, in every case of diminished or irregular menstruation. We are happy to find Dr. Dewees coincide with this opinion, which is also supported by Dr. Hall. The rule of our present author is, never to interfere unless there be some evidence that the health is suffering by the absence of this discharge. If we have to contend with obstinate and prejudiced parents, it may be prudent to appear to do something "to bring down the courses." Our opposition to their view of a subject, concerning which every woman fancies herself a very competent judge, will be unavailing and injudicious. By apparent compliance, we shall have the opportunity of treating the patient according to our own opinions.

When emmenagogues are required, Dr. D. gives frequently the volatile tincture of guaiacum, which he states never to have failed in his hands, when given in proper cases. "He exhibits it with a confidence he attaches to no other medicine for this purpose. This confidence is the result of very many years' experience of its efficacy." The mode of using this medicine is a teaspoonful three times a-day, in a little sweetened milk. The dose must be gradually increased where a perseverance beyond four or five weeks becomes necessary. Should the medicine disturb the bowels too much, a few drops of laudanum must be added to each dose. Having heard that some of his brother practitioners have not been equally successful in their employment of the guaiacum, Dr. D. observes—

"I think I can readily account for their failure: first, from their not placing the system in a proper situation for its use; and, second, by not properly persevering in the remedy. Neglecting these important points, it can readily be imagined that it may not succeed; for I deem an attention to them essential to its success, more especially in those cases where many months of interruption have existed. I think one of its superiorities consists in its certainty in cases of very long standing; and I could readily furnish from my note-book a number of instances where it succeeded to restore the menses after an interruption of from nine months to nearly three years." (P. 80.)

Another plan, which we believe originated with the French physicians, has frequently been found successful in this country,—namely, the injection of liquid ammonia into the vagina. Dr. Hosack (New-York Medical and Physical Journal,) has recorded a case of ten years' standing, which he cured by this means, after many others had been employed without benefit. He directed a drachm of ammonia, in a pint of rain-water, to be thrown up the vagina three times a-day. The cure was effected in three weeks.

In cases of dysmenorrhœa, Dr. D. recommends camphor in scruple doses; or even larger quantities, if the sufferings of the patient are not relieved. We have frequently given this medicine in such cases in our own practice, and generally with decided advantage, even after the unsuccessful administration of opium, belladonna, &c. &c. It has always, however, been our custom to employ the warm bath at the same time. The Secale Cornutum has not been very serviceable in the hands of our author in dysmenorrhœa, although it has been strongly recommended by some of his countrymen. The radical treatment of this distressing disease may be confided to the volatile Tincture of Guaiacum, paying the same regard to the state of the system as is advised when it is given in suppressed menstruation.

Dr. Dewees has seen a few cases where there was very painful menstruation without the formation, or at least the discharge, of the membranous substance which is generally found in this disease. We believe such instances are rare.

"Does this disease reside in the ovaria, or in the secreting surface of the uterus? I believe in the latter; and that its being unfavourable to impregnation is not owing to any influence it may exert upon the ovaria, (for I have reason to believe that ova have been impregnated, but, not finding the uterus in a condition to receive them, have perished,) but to either the non or the imperfect formation of the decidua. I believe the same surface furnishes both the menstrual secretion and the efflorescence called the decidua: it would seem then to follow, if it performed the first of these offices imperfectly, it would also the latter, and consequently the ovum would perish for want of a proper nidus.

"This opinion is strengthened by the facts, that, so soon as this wrong action is changed, the woman is instantly capable of being impregnated,—or, in other words, fecundation becomes successful; and also, by the influence of camphor, a temporary change is induced in the secerning vessels of the uterus, and the formation of membrane is prevented. Were it necessary, I could illustrate both of these positions by very many cases, but I shall confine myself to one of the former, as it is the most remarkable I have met with.

"In 1791, I was applied to by a lady who had always suffered at her menstrual periods, and who at such times discharged a number of membranous portions. She had been married nineteen years without being impregnated. After due preparation, for she was very plethoric, I put her upon the use of the tincture of guaiacum: in this she persevered for three months. The first period after she commenced the use of this medicine was one of prodigious severity, so much so as to make her resolve to abandon it. I, however, persuaded her to persevere: the next period was better, and the one after was without pain. She conceived immediately after, and was delivered in due time of a fine girl. She took twenty-four ounces of the tincture. (P. 91.)

In reference to this disease, we may observe, that where mucous membranes labour under a certain degree of irritation, a portion of lymph is often thrown forth with the morbid secretion that takes place on the surface, and the result is the formation of a new membrane, or membrane-like substance, which lines the cavity to a greater or less extent. The nature of this substance is regulated by that of the organ in which it takes place.* A case has been related by Dr. ROBERTSON,† in which the protrusive agony was so severe during the efforts to expel this adventitious membrane, that retroversion of the uterus took place.

We perfectly agree with the author that women are not more obnoxious to disease at the period of the decline of the menses, than at any other part of their existence. The popular prejudice upon this subject is well known, and perhaps even some members of the profession may differ from the opinion we support. Coincidences in the human system are common, and are frequently mistaken for cause and effect: hence the cessation of the menstrual discharge, and the appearance of schirrus and cancer, are considered as cause and effect.

Passing over the chapters upon *Menorrhagia,* the *Diseases of Pregnancy,* and *Displacements of the Uterus,* which the student will however peruse with much advantage, we come to the subject of *Leucorrhœa,* in the treatment of which so much difficulty is frequently experienced. It is very true that this disease is ranked by many among the *opprobria medicorum.* A variety of causes may dispose the uterus and vagina to take on the leucorrhœal action, but the production of the complaint requires an immediate exciting cause, and that cause must be of an irritating kind. Dr. Dewees divides this disease into—first, the leucorrhœa of direct irritation; second, the leucorrhœa of indirect irritation; third, the leucorrhœa of habit.

* GOOD's Study of Medicine, vol. v. p. 49.

† Edinburgh Med. and Surg. Journal, No. 73.

"Under the first head, or the leucorrhœa of direct irritation, we would consider all such instances of this discharge as follow an active inflammation of the mucous membrane of the uterus or vagina, and produced by some local cause, as laborious parturition, application of instruments, excess of venery, irritating substances applied to the surface of the vagina, extraneous bodies introduced into it, a prolapsed uterus, tumors within the vagina, injections of too stimulating a kind, or from the simple inflammation of the parts; for any portion of the body is liable to such attacks, without our being able to determine why this or that part has been selected. We have known, in a number of instances, a leucorrhœa to follow a lingering or tedious labour, both where instruments were used and where they were not, &c.

"Under the second head, or the leucorrhœa of remote or indirect irritation, we would range all such instances in which the vagina sympathises with some other portions of the body,—as with the uterus during pregnancy, or with it in long-obstructed menses, producing or becoming what is called chlorosis; with it when the menstrual action is about to furnish the catamenial discharge, or after that action has just ceased. With the rectum, when subject to hemorrhoids, or when irritated by ascarides; with the gums, as in early dentition; with the stomach, when dyspeptic, &c.

"Under the third, or leucorrhœa of habit, we would enumerate those instances of this discharge which continue after the active or inflammatory condition of the parts has ceased, as after syphilis or gonorrhœa has been cured, a prolapsed uterus restored, or a tumor removed, which may remain after the inflammation in the two former species have ceased, and the discharge become analogous to the gleet of the male. Almost every part of the body which is susceptible of action may have it to continue after it has been once excited, though the exciting cause be removed: in the nervous and muscular systems we witness it, as in chorea, whooping-cough, &c.; in the vascular and glandular systems, as in the continuance of spitting, after the action of mercury has ceased; in the membranes and vascular systems; the discharge of mucus after dysentery; and, agreeably to Mr. Hunter, as in the gleet after gonorrhœa. He distinguishes the condition of the mucous membrane of the urethra in gonorrhœa, and in gleet, in the following manner:—'The venereal inflammation is of such a nature as to go off of itself, or to wear itself out; or, in other words, it is such an action of the living powers as can subsist but a given time. But this is not the case with a gleet, which seems to take its rise from a habit of action which the parts have contracted, and, as they have no disposition to lay aside this action, it of course is continued; for we find in those gonorrhœas which last long, and are tedious in their cure, that this habit is more rooted than in those which go off soon.''—*Treatise on the Ven. Dis.* art. Gleet. (P. 226.)

We perfectly coincide with the author in the opinion that *fluor albus* has its seat for the most part in the vagina, and that it is almost always local; but, from the excess of quantity, or peculiarity in the quality of the discharge, the system sometimes becomes involved.*

The treatment of leucorrhœa should be commenced by directing that the parts be regularly washed with warm water three or four times a-day. If the patient is plethoric, the author purges her freely, confines her to milk and vegetable diet, and sometimes orders her to lose blood. The tincture of cantharides is then given in thirty-drop doses three times a-day, increasing the dose every third day, five drops at a time, until strangury is produced, unless the disease is previously arrested. Our want of success in the application of this remedy may probably have arisen from the weakness of the preparation employed. The tincture Dr. D. employs is "fifty per cent. stronger than the ordinary tincture of the shops." Astringent injections are employed as soon as a change is observed in the discharge, "by its becoming thinner and more abundant, but never until then. The best kinds of astringent injections are the acetate of zinc, in the proportion of five or six grains to the ounce of water, or the sulphate of copper in solution, in the proportion of a scruple to half a drachm in eight ounces of water: either of these may be employed three times a-day, taking care to wash out the vagina with soap and water previously." (P. 240.) Without the utmost attention to cleanliness, relief must not be expected even from the most judicious plan of treatment.

Dr. Dewees states that he has never seen an instance of the first stage of this complaint being attended by such a degree of debility, as would require either tonics or animal diet for its relief. Upon this point he is at issue with Mr. Clarke.

The second and third stages of the disease are but aggravated degrees of the first, and consequently the duty to be performed by the physician is yet more difficult. Dr. Dewees does not coincide with the opinion that it is hazardous to stop this complaint; nor have any circumstances fallen under our own notice which would induce us to believe it is well founded. It must be remembered that we rarely, if ever, can arrest the discharge suddenly. The system, therefore, becomes prepared for the change. The balsam of copaiba has

* Some cases have occurred in the practice of the Reviewer, which induce him to believe that secondary symptoms, resembling those which are consequent upon *syphilis*, sometimes arise from long-continued fluor albus, particularly in negligent and uncleanly women.

occasionally been found useful when other remedies have failed.

We believe, with Dr. Dewees, that, at the *commencement* of leucorrhœa, there is usually sufficient evidence either of inflammation of the neck of the womb or of the mucous lining of the vagina, and that consequently an antiphlogistic plan will be demanded. But it rarely happens that the practitioner is consulted until the disease has subsisted for a considerable period. He will frequently find his patient much debilitated by its continuance, and without any local or general symptoms that would contraindicate the immediate use of nourishing diet and tonic remedies. Without any wish, therefore, to countenance the inconsiderate and empirical practice of always having recourse to the stimulating plan, from the vague notion of some original local weakness, we believe that it will frequently be employed with advantage.

*Puerperal Fever.*—In the account given of this disease, the author confines himself to that inflammation of the peritoneum which succeeds delivery. He is of opinion that "this will embrace the low malignant fever of lying-in women, as detailed by Dr. Clarke, as well as the disease described by Hulme, Kirkland, Leake, Denman, Gordon, Armstrong, Hey, &c. It is true that several of these include in their accounts what they term an inflammation of the uterus,—as Hey and Denman; yet the simple inflammation of the uterus is a very different disease from puerperal fever; so much so, in our opinion, that they should never be confounded, and for this reason we have given them a separate consideration." (P.370.)

We have ourselves heard much of the distinction between peritoneal inflammation and puerperal fever, and we have earnestly sought information upon the subject from the most respectable authorities who maintain that such a distinction does exist. But we have not succeeded. No diagnostic marks have ever been described to us, or pointed out in practice, which we conceive establishes a difference between these two diseases. The different descriptions of the disease which have been given by different authorities must not be too hastily presumed to demonstrate a real distinction. They arise from the disease having been witnessed and described in its various stages. He who has seen the disease in its first stage has not hesitated to assert its inflammatory nature; while he who has been called in at the decline of the malady has lost sight of its essential character, and has applied the terms putrid, typhoid, low malignant fever, &c. and has shaped his doctrines and his practice accordingly.

"We are told that it is of the utmost consequence to the cure of this disease, that we distinguish between the true inflammatory and the putrid puerperal fever. This would be most true and important, did such a difference really exist. But the distinction attempted has been based,* we believe, upon the violence of the complaint, at different times, and under different circumstances, rather than upon any essential difference in the absolute nature of the disease." Dr. Dewees passes in review the leading doctrines of most British writers upon the subject of the puerperal fever, and does not materially differ from the opinions now generally entertained by us, either with respect to the nature or treatment of the disease.

*Milk Abscess* is frequently a source of much suffering to the patient, and great perplexity to the practitioner. Dr. Dewees says, he "has never found any application so successful in the very early stage of this disease as the frequent application of warm vinegar to the part. Its efficacy appears to us so certain, when sufficiently soon employed, that we need not in many instances look for any other remedy. It is particularly prompt in that condition of the breasts in which a want of proper drawing leaves them, or where they become suddenly and painfully distended by the sudden secretion of milk, but which cannot be extracted with ease, or in sufficient quantity, either from a defect in the nipples, or in the external or inferior extremities of the tubuli lactiferi." (P. 463.) The vinegar should be employed very perseveringly for at least twenty-four hours. If the pain and intumescence be not then abated, leeches should be applied in sufficient number to abstract from ten to twelve ounces of blood. We are to persevere in the leeching until we cannot hope to prevent suppuration.

"When this happens, let it be remembered we are not to promote the tendency to suppuration by poulticing, &c. for this only increases the pending mischief by the formation of a greater quantity of pus, and the consequent destruction of a greater quantity of the substance of the breast, by which its future usefulness may be entirely destroyed. It should therefore be a never-failing rule to treat a mammary inflammation as if it would not suppurate.

"From the period in which we look with certainty for the breast to suppurate, to the time at which this takes place, some saturnine application should be employed steadily. We are in the habit of using the following liniment for this purpose, and we think with advantage:—℞. Ol. Olivar. Opt. ℥ij.; Liq. Plumbi sub acetatis,

* It would be affectation to deny that we understand the meaning of the word "based," as it is here employed. The only meaning, however, of the verb "to base" is to embase—to degrade.—R.

ʒj.; Æther. Vitriol. ʒij., Tinct. Thebaic. ʒj. M. A rag to be moistened with this liniment, and applied to the part frequently.

"Dr. Clarke speaks highly of the following formula, for the same purpose:—℞. Cerussa Acetata ʒj.; Acetum Distil. ℥ij. f. sol. adde Sp. Vin. rect. ℥j.; Aq. Distil. ℥v. M. This is to be applied constantly to the breast, cold, by means of a piece of linen. By this plan we prevent the formation of an over quantity of pus; we preserve the integrity of the external covering of part; and we prevent an œdema, almost certainly. (P. 464.)

Of the application of vinegar in the manner recommended by our author, we know nothing from our own experience. We have usually employed a tepid spirituous lotion. We shall certainly take an opportunity of adopting the remedy he so strongly recommends. We know not how tractable the American ladies may be to the advice of their physicians, but we confess we have had much difficulty in overcoming the popular partiality for the constant application of hot poultices in the treatment of milk abscess, although in most cases their continued employment is decidedly injurious. If matter forms, the breast must be treated upon common principles.

"After the healing of this abscess, a considerable hardness remains in the breast, which will require a long time for its absorption: this creates a good deal of uneasiness in the patient, lest it be a scirrhus. On this point her fears may be composed, as the tumor which remains will eventually be taken up by the absorbents, as it is nothing but coagulated lymph; but this may be promoted by keeping the part warmly covered, or by the repeated application of vinegar. If this occur in winter, a piece of rabbit-skin may be used, with the furred side next to the breast; if in summer, a piece of fine flannel will answer very well: or the part may be rubbed twice a-day with opodeldoc." (P. 467.)

The volume concludes with a chapter on *Hysteria;* in which, however, we find nothing of sufficient novelty to require any comment.

We have upon several occasions offered our mite of praise to the talents of Dr. Dewees: the work we have just noticed affords an additional proof of his practical experience and discrimination.

---

*Das Saugadersystem der Wirbelthiere.* Von VINCENZ FOHMANN. Erstes Heft. *Das Sangadersystem der Fische.* Mit 18 Steindrucktafeln.—Fol. pp. 46.—Heidelberg und Leipzig, 1827.

THIS is the first part of a work on the Natural History of the Absorbent System in the Vertebralia, by Professor Fohmann, recently of Heidelberg, now of the University of Liege. It treats of the absorbent system in Fishes, and is adorned with beautiful lithographic plates; the second part will contain that of Reptiles; the third, that of Birds; and the fourth, that of the Mammalia and Man.

The attention of the author has been for a considerable time directed to this part of anatomy. Six years ago he published an account* of some researches he had made on the communication between the absorbents and veins. Since that time he has steadily prosecuted the subject; all who have had an opportunity of examining the anatomical collection at Heidelberg, and seeing the beautiful preparations of injected absorbents (lacteals and lymphatics) which it contains, must know the zeal displayed by Professor Fohmann in the pursuit of his object. We may mention that he made a journey into Holland for the special purpose of obtaining more ample opportunities of investigating the anatomical disposition of the absorbent system in the class of animals, the account of which is now given to the public.

Whilst the osseous, the nervous, the respiratory, and the vascular, systems have been examined with great care, in an extensive series of animals, the absorbent system, comprehending under this name the lacteals and lymphatics, has been almost disregarded by comparative anatomists. With the exception of some imperfect descriptions of these vessels in a few of the mammalia and in the turtle, our knowledge of the absorbents in the three lower classes of animals, birds, reptiles, and fishes, has remained precisely as it was given to us by the discoverers of this system in these three classes, Monro and Hewson, more than half a century ago. Blumenbach, in his Manual of Comparative Anatomy, merely repeats what they have said; Cuvier does the same, and candidly owns that he has made no addition to this part of comparative anatomy. "Nous ne pourrons en donner, au reste, qu'une anatomie comparée fort succincte, et en grande partie empruntée de nos prédécesseurs, parce que nous n'avons fait, par nous mêmes, qu'un petit nombre de recherches sur cette branche de la science." (vol. iv. p. 93, *Des Vaisseaux et des Glandes Lymphatiques.*)

The discovery of absorbents in the three classes of animals above mentioned excited much attention at the time, and a very warm controversy between the two celebrated individuals who respectively claimed the merit of it. The controversy, however, seems to have excited more interest than the subject of discovery; for, instead of its being followed up by the

* Dr. V. Fohmann, Anatomische Untersuchungen über die Verbindung der Sangadern mit den venen; mit einer vorrede Von Fridr. Tiedemann.—8vo. Heidelberg, 1821.

investigations of succeeding anatomists into the anatomical disposition of the absorbent system in other animals than those in which its presence had been ascertained and described by Monro and Hewson, so entirely did the interest cease and the matter fall into abeyance, that a physiologist of the present day has raised a question whether there was any truth in the discovery at all. M. Magendie has presumed to state it as his opinion, "that, although all anatomists since Hewson and Monro have acknowledged that birds, reptiles, and fishes have a chyliferous apparatus, yet he doubts it; and that, referring to his own dissections, he should say that the mammalia and some reptiles alone have a chyliferous apparatus, and possess chyle."

To discover and to inject the absorbents in fishes, are not matters of immediate facility to those not familiar with the subject: it requires great perseverance, considerable practice, and some manual dexterity, to accomplish these objects. We do not enter into the nature of the difficulties, nor the means of overcoming them; but we may mention that, although our author was not long in making himself acquainted with the absorbents of reptiles and birds, yet for some time he was not so fortunate with regard to fishes. It was in vain that he attempted to discover them in the animals of the class which inhabit the rivers in the vicinity of Heidelberg, the Rhine, and the Necker. To attain his object, he found it necessary to go to the sea-coast, in order to make the attempt on fishes of larger size. For this purpose he went to Leyden, where he was kindly accommodated with a room in the Zoological Museum, by Dr. Temminck, to whom, with Dr. Boie, he expresses himself grateful for the assistance they afforded him in his pursuits. The first animal he received was an individual of the species Silurus Glanis, and in which he had succeeded in injecting the arteries, veins, and absorbents. He then with less difficulty discovered the absorbents in the torpedo, and soon succeeded in recognising and injecting the small absorbents of the turbot. Having thus acquired some familiarity with the subject, he did not abandon the hope of being able to discover the same vessels in those species of fish in which he had previously so long sought for them in vain; and, although even now he was not invariably successful on the first attempt, yet, after repeated trials, he succeeded in inflating some twigs or branches, and thus paved the way, from the want of valves in the absorbents of fishes, for their further successful injection.

How much these patient labours, for labour it must be considered, of the German anatomist may be adapted to the character of mind of the great French Vivisectionist, may perhaps admit of question; but, as we have alluded to a declaration of the latter, we extract from the introductory chapter of the former the following paragraph in relation to it:

"How erroneous is the assertion of Magendie, that the absorbents are confined to the superior classes of vertebralia, I have fully convinced myself. Not only have I seen and examined these vessels in the animals in which their presence had been ascertained and descriptions given of them by Monro and Hewson, but I have discovered them in many other orders and species, in which their existence had merely been assumed as probable. I have repeatedly filled the blood-vessels in individuals of various orders of fishes, amphibiæ, and birds, with coloured materials, and, after the most successful injections of the arteries and veins, there still every where remained other vessels, having all the characters of absorbents, and which, on the intestinal canal, I frequently saw containing chyle. The number of these vessels on the organs of digestion is innumerable, they envelope them on all sides, and usually form superficial and deep-seated plexuses, which in the different classes and orders are differently arranged." (P. 9.)

The individuals of the class of Fishes whose absorbents have been described and represented by Monro, are the skate, haddock, cod, and salmon. Hewson has given a description of the absorbents of the haddock, and, when he comes to those of the intestinal canal, he alludes to the cod, the plaice, and the skate. Professor Fohmann has added greatly to this list, and has examined the subject with more accuracy. His descriptions are taken, and conclusions drawn, from observations on those of the torpedo, the sea-dog, the eel and sea-eel, the pike, the silurus glanis, the sea-wolf or sea-cat, the turbot, the cod, the burbot, the salmon, and the lophius piscatorius. The engravings which he has given are from drawings of those of the torpedo marmorata, the eel, the pike, the silurus glanis, the turbot, the sea-wolf, the cod, and the salmon: they are beautiful representations of the arrangement and disposition of the absorbent vessels in these animals, more especially those of the organs of digestion, and they have a character of truth about them which rendered unnecessary the confirmation of a friend who has seen the preparations from which the drawings were taken, that they are faithful and accurate representations of nature. The author does not pretend that his publication is in any way to be regarded as a complete work on the subject, seeing it treats of the absorbents in but a small number of fishes, yet he has exerted himself to the utmost of his opportunities; and, if we bring the individuals above mentioned into systematic arrangement, we shall find that the genera Raia, Squalus, Muroena, Esox, Pleunorectes, Silurus, Anorrhichas, Gadus, Salmo, and Lophius, constitute a considerable number of orders and species in which the absorbents have been examined, and in which we find them differing almost as much from each other as the species of this class of animals differ.

We cannot enter into a full account of M. Fohmann's researches, but there are one or two points to which we shall venture to call attention. In the first place, then, with regard to the termination,—or we should rather say, the commencement,—of the absorbents, he is at variance with Monro and Hewson, who state that it is by open mouths; and, after giving good grounds for doubting the accuracy of the observations of the former, and showing the fallacy of the experiments of the latter, he states that he never could discover open mouths in the absorbents of the torpedo and sea-wolf, in whom those of the mucous membrane of the intestinal canal can be seen and accurately examined by the naked eye; and as little could he find them in those of other species of this class of animals. He states that these vessels in fishes invariably terminate in blind extremities, *en cul de sac,* at this place constituting tubes of greater diameter than they do a little further from their commencement,—i. e. in their course towards the centre of circulation; and that, in most parts of the body, they here represent pouches or dilatations, which have an internal smooth surface, and an external one having more or less resemblance to the appearance of cellular substance.

As regards the fluid corresponding to that which we are accustomed to find in the absorbents of the digestive organs of the mammalia during the process of chylification, Professor Fohmann states that, although he has directed his attention particularly to this point, he has not often met with it in fishes, probably from the circumstance of digestion and the absorption of the chyle having already terminated when he received the animals, or because the quantity of fat which frequently surrounds the absorbent vessels did not allow the fluids they contained to be recognised. "Still, (he proceeds,) in several instances, I found the nutritive fluid (*Nahrungs saft*) in the absorbents of the living torpedo; it had not the milky appearance of chyle in the mammalia, nor was it transparent, but had a greyish colour." (P. 43.)

It is known that in fishes the absorbents have no valves, with the exception of the places where they terminate in the venous system, and also that there are no absorbent glands.

These vessels, however, do not preserve a continuous vascular character throughout their course. Having preserved this for a short distance, they suddenly increase in size, run together, and either constitute or terminate in sacculi or plexuses. The internal surface of these sacculi or plexuses does not present a smooth surface, but a rough irregular one of filamentous, membranous, or cellular substance-like projections; so that what externally appears as one simple sac, is divided internally into several by these lamellæ (*blätchen*). This is readily seen in the absorbent sacculi (*sangadersäcken*) in the vicinity of the stomach and intestinal canal of the eel, and in the absorbent plexuses (*sangadergeflechten*) of the stomach of the torpedo. These absorbent sacs and plexuses, which are not confined to the class of fishes, but are met with in the amphibiæ, are parts well worthy of notice and consideration.

On the subject of the communication between the absorbents and veins, our author observes, that those who have assumed such a communication to exist, do not seem to have bestowed sufficient time on their investigations, and have not gone to work with the caution and precision which this very difficult subject requires. He instances the observations of Lippi* as being particularly liable to this objection, and not at all to be depended on; in truth, they are full of error. The observations of the Italian are asserted to have been founded on examination of the absorbents in man, the horse, and the goose. He represents a connexion as existing between the extremities of the absorbents and extremities of the veins, like that between the capillary arteries and veins, the one kind of vessel passing into, or rather continuing itself into the other. Fohmann, after the most numerous and successful injections of the absorbents in man and the mammalia, has never seen this communication. The circulating system forms a shut circle; the only communication between absorbents and veins is that where the absorbent opens through the coats of the vein, *insertio lateralis;* all communication between capillary vessels is *insertio terminalis,*—the transition of the extremity of an artery into the commencement of a vein. Lippi has represented the absorbents as communicating by means of greater or smaller twigs, branches, and trunks, with the venous system, and that out of the limit of the absorbent glands, those twigs, branches,

* Illustrazioni Fiscologiche del Systema Linfatico-chilifero Mediante la Scoparta di un gran summo di Communicazioni di esso col Venoso; del Prof. Regolo Lippi.—Frienze, 1825, 4.

and trunks opening directly into the vena cava, the internal pudic and emulgent veins, and the vena azygos. Fohmann asserts that, in man and the mammalia, a communication between the veins and absorbents only takes place in the absorbent glands; and that none exists by means of such large vessels, or out of the absorbent glands, as stated by Lippi, who, in fact, has taken veins for absorbents. In the animals in whom absorbent glands do not for the most part exist, in fish, amphibiæ, and birds, we can observe the communication between the absorbents and veins, in different parts of the body, with the naked eye. The absorbents which Lippi represents as proceeding from the under part of the intestines of the goose into the renal vein, are the vessels of whose connexion with the renal or sacral vein Fohmann gives an account in his small work published six years ago.

A curious circumstance discovered by our author in certain fishes, is that the contents of the absorbents are not all poured directly into the venous system. In the eel, for instance, a branch goes off from the trunk of the absorbent system, representing the thoracic duct, towards a small receptacle in the vicinity of the gills. Out of this globular receptacle proceeds a vessel, which divides into branches; these branches are distributed to the gills, in the same way as those of the pulmonary artery, and from their extremities are returning vessels, which bring back the lymph, in the same way as the pulmonary veins return the blood; the trunk formed by these branches opens into the thoracic duct immediately before its termination in the jugular vein.

We shall conclude our notice of the work by giving the author's view of the mechanism of absorption.

The loose tissue covering the external surface of the origin of the absorbents establishes a connexion between them and the other systems of the body. It forms, as it were, a sort of sponge around them, which, exercising an absorbent power on the matters susceptible of being absorbed, conducts them to the thin parietes of the vascular system, through which they pass. It is not to the fluid that the tendency to penetrate our tissues is to be attributed: on the contrary, it is attracted by those parts whose structure renders them capable of exercising attraction. This act of absorption, then, by the animal tissues, although not an effect of vitality, is indirectly under the influence of the vital powers, as the progress of the fluid, once within the lymphatic vessels, depends on the contractility of living parts, without which progress the continuance of absorption could not exist; just as sponge, once filled with fluid, ceases to attract more until it has been squeezed, and thus reacquired the power of absorbing. That the absorbents have the power of contracting on themselves, Professor Fohmann satisfied himself by observation in the living ray, where, on opening the abdominal cavity, he found the lacteals less than when the animal had been some time killed, and where, on injecting air or other fluid into them, they did not dilate to the same extent as when the injection was made after death.

---

*A Physiological Enquiry respecting the action of Moxa, and its utility in inveterate cases of Sciatica, Lumbago, Paraphlegia, Epilepsy, and some other painful, paralitic, and spasmodic diseases of the Nerves and Muscles.* By WILLIAM WALLACE, M. R. I. A., &c. &c., Surgeon to the Charitable Infirmary of Dublin, and to the Infirmary for the Treatment of Rheumatism and Cutaneous Diseases in that city, Lecturer on Semeiology and Clinical Surgery. 8vo. pp. 148. Hodges and M'Arthur. Dublin, 1827.

FROM works like the present we generally expect satisfactory information. Compilations upon a variety of subjects are of course highly necessary as books of general reference, but if we wish to form a correct estimate of the power of a single remedy, whose character is yet to be established, we must look to those who have particularly devoted themselves to determine how it is to be applied, and what effect it is capable of producing. As the rays must converge to a point, in order to glow intensely, so is it more probable, that he, who for a time concentrates his attention upon one mode of practice, will form a more correct view of its merit than if he had directed the current of his thoughts to a great variety of subjects, and had endeavoured rather to reconcile the discrepancies of others than to form opinions for himself.

Whatever may be the reputation which the Moxa possesses among the continental physicians, in this country its application has been too limited to have gained for it any decided character. A few of our practitioners, indeed, have highly vaunted its powers; but others have declared that it can seldom claim any superiority over numerous much milder counter-irritants, and some have gone so far as to assert that its only peculiar effect is the excessive pain it produces.

It must be desirable, therefore, that the subject should be fully discussed, in order that we may be enabled to determine the rank which moxa ought to hold in our list of remedies. Mr. Wallace has dedicated his work to the Baron Larry, "to whose writings the moxa is indebted for its present rank on the Continent of Europe." We read the Baron's memoir upon this subject with much attention, and the impression it left upon us was, that he had lavished his praises too indiscriminately. When we are called upon to believe that either men or medicines are capable of doing every thing, we cannot but suspect that the partiality of the eulogist has somewhat obscured his better judgment. The extent of Baron Larry's confidence in the moxa may be imagined from the assurance with which one of his chapters commences, that "in *all* chronic affections of the head he has employed this remedy with the greatest success."

The moxa, we believe, has been hitherto regarded, in this country, as a mere counter-irritant, not essentially differing in power from other remedies of the same class. Mr. Wallace is anxious "to demonstrate *in limine* that the prevailing opinions repecting the mode of action of moxa are completely erroneous; and to show that this agent affords a remedy which cannot be replaced by any other yet known." "If, says Mr. Wallace, it should be asserted that the moxa has not fulfilled the expectations in this country, which have been raised by continental practitioners, the following is my answer, —that as far as I have heard of its employment in these countries, it has been used upon no fixed principle at all, or upon an erroneous one; cases have been selected for its application without any scientific ground of choice; and in no one instance have I heard of its proper employment having been persevered in, so as to afford a rational hope of benefit."

The excessive pain which this remedy is generally supposed to inflict is doubtless a great obstacle to its more frequent employment. Our author has had many opportunities of ascertaining the opinions of patients upon the comparative pain produced by moxa, caustic issues, and blisters, and he has "never met with a single instance in which the moxa, when properly applied, has not been considered the mildest remedy by many degrees."

Having premised some introductory observations for the purpose of preparing the reader for an unprejudiced investigation of the facts and conclusions which are to follow, Mr. Wallace proceeds to offer some reflections on the nature or immediate cause of those morbid states, which the moxa is said to cure or relieve, in order that we may not make an unscientific application of the remedy. He thinks that the distinction usually made between *functional* and *organic* disease is inaccurate, — but in this we cannot agree with him. Judging from the line of argument he adopts upon this subject, we infer that he leaves entirely out of the question the various changes and modifications of the vital principle, from which functional derangement so frequently results without organic lesion. An organ may have entirely lost its power of performing its appropriate function, and yet present precisely the same appearances as if it had been capable of fulfilling the task which nature had assigned to it in the animal economy. We here must presume that some property must have been lost which was independent of its organization. It would be very easy to adduce many examples, to show how frequently functional derangement exists without organic lesion. For instance, a man sits down to his meal with his stomach just in tune for a hearty repast. He suddenly receives some powerful moral impression, the vital principle of the stomach is consequently deranged, and it is no longer capable of performing its function. The appetite has vanished and a sensation of sickness comes on. Here there is sufficient evidence of functional disturbance, but it will not be urged that any organic lesion of the stomach has taken place, for the moment the mind has recovered its tranquillity the stomach is again equal to perform its duty.

*Of the mode of action of Moxa.*—The author correctly observes that the effects of moxa are generally compared to those which result from blisters or issues.

"Nothing can be more unfounded than such an opinion. Of this the reader will be convinced hereafter. In the mean time, it may be observed, that the good effects of a blister are attributable, partly to the lacal inflammation or counter-irritation excited by it, and partly to serous or purulent discharge which results: while the action of an issue depends on the purulent drain which it causes. Whereas, the Moxa, when properly applied, and in appropriate cases, may be said to produce neither inflammation nor discharge. In fact, if it should excite much inflammation, it must be quite certain that the Moxa is either not suited to the case, or that it has been incorrectly applied; and, on many occasions, no discharge whatever results. But, even when a discharge does take place, the good effects of the Moxa have occurred, long before the dischage has been established; and, although the discharge may be useful in some particular cases, its beneficial effects depend upon a principle altogether different from that which explains the utility of Moxa. This remedy, therefore, does not act either as a blister or issue; and it will be proved in the sequel, that the practitioner, who shall regulate his conduct as if he expected such effects, must inevitably fail, in obtaining the beneficial influence of Moxa, in nine cases out of ten in which he may employ it.

The experiments of Drs. Philip and Hastings are referred to, and some observations added by the author himself, from which he thinks the conclusion fully authorized, "that the application of a certain degree of caloric to the living body, is followed by a *contraction of the capillaries*, and an *increase in the rapidity of their circulation*; and that this contraction does not proceed from a mere *physical* action, but is the result of the influence of the heat on the *vital properties* of the vessels."

In case it should be presumed that the influence of caloric is only temporary, the author made some experiments on his own person for the purpose of showing that its influence persists after the period of its application; and therefore that it is likely to afford permanent assistance in controling or removing diseased action. We would suggest, that the effect which caloric produces upon the animal economy, whether locally or generally applied, are not positive; they will be found as fluctuating as the vital powers of the patient to whom it is applied.

The direct effects of Moxa, in the opinion of Mr. Wallace, are not confined to the skin.

"If an eschar be formed, that eschar extends to the superficial fascia or subcutaneous cellular tissue: and if the Moxa be applied through the medium of a needle, in the manner which I shall hereafter describe, the caloric may be made to extend its influence as deep as we please, by the conducting power of the needle. If the caloric, by either mode of application, be made to act on a point of structure, which extends itself indefinitely through the limb or part of the body in which it is seated, as for example the cellular tissue, or the neurileme of a nerve, or the tunic of a vessel, it is perfectly consistent with our knowledge of the mode of action of such remedies, to suppose that its effects may be extended to any length along the texture so acted on.

And, "when the moxa is properly applied there is scarcely any inflammation excited, and on the other hand, its beneficial influence occurs and has terminated long before a discharge is established."

The author having shewn that the remedy acts on the capillaries and absorbents as a local tonic or stimulating astringent, energetic and continued in its influence, it follows, in his opinion, "that the moxa should be employed in those cases only in which there exists a state of debility of the capillaries, a consequent retardation of their circulation, and a diminution of absorption, and that it must be injurious if employed in cases where there is increased action or active inflammation." The liberality and candour with which Mr. Wallace confesses that "misapplications of a similar kind" occurred in his own practice, before he had formed a correct opinion respecting the mode of action of the remedy, do him much honour.

In cases to which blisters are peculiarly suited, moxa frequently does more harm than good. The following maxim is not to be deviated from, "that this remedy shall never be employed in cases of increased action, or of active inflammation, or even in cases of sub-acute inflammation; that is, when the acute inflammation is lapsing into the state of chronic action; and this principle should be implicitly adhered to, whether the active inflammation has attacked parts previously in a state of health, or has supervened on the state of passive inflammation."

Mr. Wallace very properly urges that it is better to err by continuing the employment of those remedies which are suited to lower increased action, than to conclude incautiously that all inflammatory action has passed by; and thus too soon have recourse to a remedy which would in all probability aggravate every bad symptom, and give unnecessary pain to the patient. Some useful practical hints are offered to assist our diagnosis between the opposite states of chronic and acute inflammation. Aware of the difficulty of properly applying evaporating lotions, Mr. W. gives a description and plate of an instrument which appears ingeniously contrived to ensure the continued and effectual application of such remedies. The instrument supplies no more than the exact quantity of fluid which is carried off by evaporation; and consequently there is none allowed to pass to the bed-clothes.

*Of the various modes of applying Moxa.*—By different practitioners a great variety of substances have been employed to cause "moxabustion." In those countries in which moxa is much employed, and its value known, it has been the universal custom to attribute a portion of its medical efficacy to some peculiar volatile substance disengaged during its application. Caloric, however, is the only agent evolved during the combustion of the various substances from which any therapeutic influence can be expected; we should select a substance whose combustion will take place slowly but steadily. If the combustion be too quick the effects will be too transitory, and if too slow it will require the use of the blow-pipe, which complicates the operation and produces unnecessary alarm. The author has been fortunate enough to discover a mode of forming moxa, which is free from both objections.

"It is formed by immersing, either surgeon's lint or fine linen, in a filtered solution of chlorate of potash: the solution being made by dissolving one drachm of the salt in four ounces of distilled water. When the moxa is to be used of a small size, fine linen will answer best, but when of a large size, lint is to be preferred. Care must be taken that the substance used shall be perfectly dry, before it be folded up, and in folding it, a proper degree of firmness must be given, which experience will soon teach. After the substance has been rolled up and fastened with two or three stitches of the needle, its end should be cut with a very sharp knife, to make it perfectly level, and thus secure its application to every part of the skin upon which it is placed. Its length should be about three-fourths of an inch, and its diameter may vary from one quarter of an inch to an inch.

The instruments which I use in applying the moxa are of the most simple kind: a porte-aguille which I have invented, or a pair of dressing or artery forceps, furnished with a screw at about three-fourths of an inch distant from their point, which screw serves to press the blades of the forceps very tightly together;—a bit of small, flat, silver wire, about three inches in length;—a bit of card paper;—a blow-pipe; a set of needles; and a small glass tube, are all that are required. With the silver wire a small hoop is formed to grasp the moxa: the size of the hoop being made to vary according to the size of the moxa; and the ends of the hoop are grasped in the forceps, which are made tight on it by the screw with which they are furnished. The hoop should be applied about a line distant from that end of the moxa which is to be placed on the skin; for the purpose of preventing any inconvenience from the hot wire coming in contact with the surface. In fixing the ends of the hoop in the forceps, such an angle or inclination of the moxa with the forceps should be given, as will be found most convenient for the exact application of the moxa to the part affected."

In painful affections, the moxa should be applied where most distress is felt. In paralytic affections it should be first applied over the origin of the nerves which lead to the diseased parts, and afterwards along the same nerves in different parts of their course. Mr. Wallace has ventured to apply it "to the eye, in the form of *objective* moxa," in some cases of obstinate chronic ophthalmia, and, it is said, with great advantage.

"The size of the moxa, the manner in which it should be applied, and the length of time it should be allowed to remain on the parts, are points of some importance.—All these circumstances must be regulated by the depth of the disease, and the nature of the parts, to which we may wish to apply it. It may be used so as not to cause any injury of texture; in a greater degree so as to produce vesication; and in a still greater degree an eschar, and the eschar may be either deep or superficial; or, lastly, it may be employed in conjunction with the acupuncture needle. These different modes of using the moxa may be distinguished by the terms, first, second, third, fourth, and fifth, forms of application."

The first form of application will answer when the disease is very superficial. It constitutes the objective cautery of the French writers. Mr. Wallace seldom has recourse to the second form of application. He considers it less effectual than the third, and it is more troblesome in its after treatment. It may, however, be usefully employed in tic doloureux, and to those parts on which the patient would not wish a cicatrix to be formed. In such cases the moxa is applied by holding it steadily, and as close as possible to the skin, without allowing it to touch it, and until the skin appears white; which appearance is owing to the detachment of the cuticle, and the formation of a blister.

"In a large proportion of cases the superficial eschar will be the best form of application. To produce this eschar, the moxa must be placed on the skin, and allowed to remain on, until the skin appears brown under it; which will, in general, be found to take place, when the combustion of the moxa has extended to the distance of about a line from the skin.

"The deep eschar will be required when the seat of the disease is far removed from the surface, as in affections of the spinal marrow and of the hip. To form this eschar the moxa must be allowed to remain on, until its combustion is complete; when the part upon which it was seated will be found black, and the surrounding skin slightly red, and wrinkled. In this form of application, it will be sometimes useful to increase the intensity of the heat by the employment of the blow-pipe; and when this is thought prudent, the moxa should be, previously to its application, surrounded by a cylinder of card-paper, which will have the effect of directing the current of heat downwards, and prevent its escape laterally."

The mode of using the moxa in conjunction with the acupuncture needle, is also pointed out. The author confirms the observation made by Larrey as to the effects of ammonia in preventing the occurrence of inflammation from the application of this remedy.

The fifth section occupies many pages, and presents a general survey "of the means which may be employed as adjuvants to moxa."

The sixth section contains "Cases of painful, paralytic, and spasmodic Diseases of the Nerves and Muscles, to illustrate the utility of Moxa." They are concisely related, and certainly afford very strong proofs of the powers of the remedy. In most of them the disease had been of long duration, and had resisted various modes of treatment, *including the usual counter-irritants,* such as blisters, setons, issues, &c. &c. We should observe also, that, in the majority of these cases, no other remedy was conjoined with the moxa. We are consequently not involved in the difficulty of estimating the power of a particular agent, which must always be the case when at the same moment many means are had recourse to.

We are of opinion that Mr. Wallace's book cannot but raise the reputation of moxa in this country. He is naturally partial to a remedy which he has frequently and successfully employed; but we do not discover, in any part of his book, that "stretch of praise" which leads to doubt rather than to confidence.

---

*Practical Observations on the Management and Diseases of Children.* By the late CHARLES THOMAS HADEN, Esq. With additional Observations, and a Biographical Notice of the Author, by THOMAS ALCOCK, Surgeon.—8vo. pp. 188. London, 1827.

THE essays contained in the present volume are republished from Mr. HADEN's Journal of Popular Medicine, which is now out of print. They are intended for the perusal of intelligent parents, and are certainly well calculated for their improvement. The directions given are clear and simple, and the style in which the author conveys his opinions is unincumbered by technicalities, which would ill accord with the avowed intention of his labours. The medical student also may gather some hints from the observations of the author, which will be of service to him in the commencement of his practical career. In common with every member of the profession, we must regret the loss of Mr. Haden: his abilities were good, and his life was entirely devoted to the improvement of his profession.

The additional observations by Mr. ALCOCK are contained in four short chapters, and treat of "Weaning, and the diseases incident to that period;—on the mode of bringing up Infants by Hand;—on the Management of Children, from the period of teething to the commencement of school education;—on Preparatory Schools, and on the Precautions and Management required to promote Health."

Upon the subject of bringing-up children by hand, Mr. Alcock states that it has been a part of his duty to endeavour to ascertain the amount of mortality among infants from this source, and, after much careful inquiry and investigation, he is convinced that the attempt to bring-up children by hand proves fatal, *in London,* to at least *seven* out of *eight*

of these miserable sufferers; and this happens whether the child has never taken the breast at all, or having been suckled for three or weeks only, is then weaned. *In the country*, the mortality among dry-nursed children is not so great.

It would have been satisfactory had Mr. Alcock stated from what class of society he had formed this distressing conclusion. The loss of children brought up by hand, under our observation, has certainly not been nearly so great.

Mr. Alcock's experience does not accord with that of Dr. Cheyne in the treatment of the bowel complaints in children: so far from finding calomel generally necessary or beneficial in these diseases, he has frequently witnessed the irritation of the bowels kept up so long as the use of calomel has been persisted. We have frequently touched upon this subject, and the opinions we have from time to time expressed show that we agree with the remarks he has offered upon it.

---

